# Blood urea nitrogen to serum albumin ratio as a prognostic marker for 28-day mortality in atrial fibrillation: a retrospective cohort study

**DOI:** 10.3389/fcvm.2025.1533575

**Published:** 2025-05-09

**Authors:** Yun Huang, Guangdong Wang, Xia Xiang, Jun Mei, Chunyan Zhang, Yaxin Zhang

**Affiliations:** ^1^ Department of International Medical Center, First People's Hospital of Foshan, Foshan, Guangdong, China; ^2^ Department of Respiratory and Critical Care Medicine, First Affiliated Hospital of Xi’an Jiaotong University, Xi’an, Shanxi, China; ^3^ Department of Nursing, First People’s Hospital of Foshan, Foshan, Guangdong, China; ^4^ Department of Neurology, Xiamen Humanity Hospital, Fujian Medical University, Xiamen Fujian, China

**Keywords:** blood urea nitrogen to serum albumin ratio, atrial fibrillation, MIMIC-IV database, mortality, retrospective cohort study

## Abstract

**Background:**

The blood urea nitrogen to serum albumin ratio (BAR) has emerged as a potential prognostic marker. This study investigated its association with clinical outcomes in patients with atrial fibrillation (AF).

**Methods:**

A retrospective cohort analysis was performed using data from the MIMIC-IV 2.2 database, including 4,977 patients diagnosed with AF. The primary outcome was 28-day mortality. Cox proportional hazards models were applied to evaluate the association between BAR and mortality, and restricted cubic spline (RCS) analysis was used to explore potential non-linear relationships.

**Results:**

Of the 4,977 patients analyzed, the 28-day mortality rate was 22.99%. Higher BAR levels were significantly associated with increased risk of mortality. Each one-unit increase in BAR was associated with a 2% higher risk of 28-day mortality (HR 1.02, 95% CI 1.01–1.03). Compared with the lowest quartile (Q1), patients in the highest quartile (Q4) had a significantly increased risk (HR 1.78, 95% CI 1.42–2.22). ROC analysis showed an area under the curve (AUC) of 0.65 for BAR in predicting 28-day mortality. Subgroup analyses confirmed the consistency and robustness of these findings across diverse clinical strata.

**Conclusions:**

BAR is an independent predictor of 28-day mortality in patients with AF. Higher BAR levels are strongly associated with worse outcomes, underscoring its potential utility as a risk stratification tool in this population.

## 1 Introduction

Atrial fibrillation (AF) is the most prevalent cardiac arrhythmia, characterized by rapid and disorganized electrical activity in the atria, resulting in an irregular heartbeat (1, 2). Despite significant progress in its diagnosis and treatment (3), the global prevalence and incidence of AF have markedly increased over the past three decades. In 2021 alone, the global incidence of AF reached 4.48 million (4). In Europe and the United States, approximately 25% of individuals over 55 years of age are projected to develop AF during their lifetime (5). AF poses a substantial public health burden due to its high rates of comorbidity, increased mortality risks, and escalating healthcare costs (6). Early identification of individuals at heightened risk for AF development is critical for effective clinical management (7, 8).

Blood-based biomarkers have shown promise in improving prognostic accuracy in AF (9). However, current tools such as the CHADS₂ and CHA₂DS₂-VASc scores are limited in individualized risk prediction. Traditional biomarkers—including troponin and natriuretic peptides—lack sufficient specificity for AF, as they are elevated in various cardiovascular conditions (10). Moreover, the cost and complexity of certain tests, such as echocardiography, can hinder widespread clinical adoption (11). Despite extensive efforts, a definitive, reliable prognostic biomarker for AF remains elusive (12).

Blood urea nitrogen (BUN) and serum albumin are well-established indicators of renal function, nutritional status, and systemic inflammation (13). Elevated BUN has been linked to poor outcomes in cardiovascular conditions (14), while hypoalbuminemia is a known predictor of mortality in critical illness, including AF (15).

The blood urea nitrogen to serum albumin ratio (BAR), reflecting both renal function and nutritional status, has emerged as a prognostic indicator in various conditions, including heart failure (16), acute myocardial infarction (17), chronic obstructive pulmonary disease (18), and sepsis (19). However, its role in predicting short-term outcomes in AF patients remains unclear. This study aims to explore the association between BAR and the prognosis of patients with AF.

## 2 Materials and methods

### 2.1 Database introduction

We conducted a retrospective cohort study using the MIMIC-IV v2.2 database, which contains de-identified electronic health records of ICU patients admitted to the Beth Israel Deaconess Medical Center from 2008–2019. The dataset includes demographics, vital signs, laboratory results, medications, and clinical outcomes for approximately 60,000 ICU admissions. The first author of this study, Yun Huang, successfully completed the required training and certification exam (certificate number: 62970244) and was granted access to the database.

### 2.2 Population selection criteria

**Figure 1:**
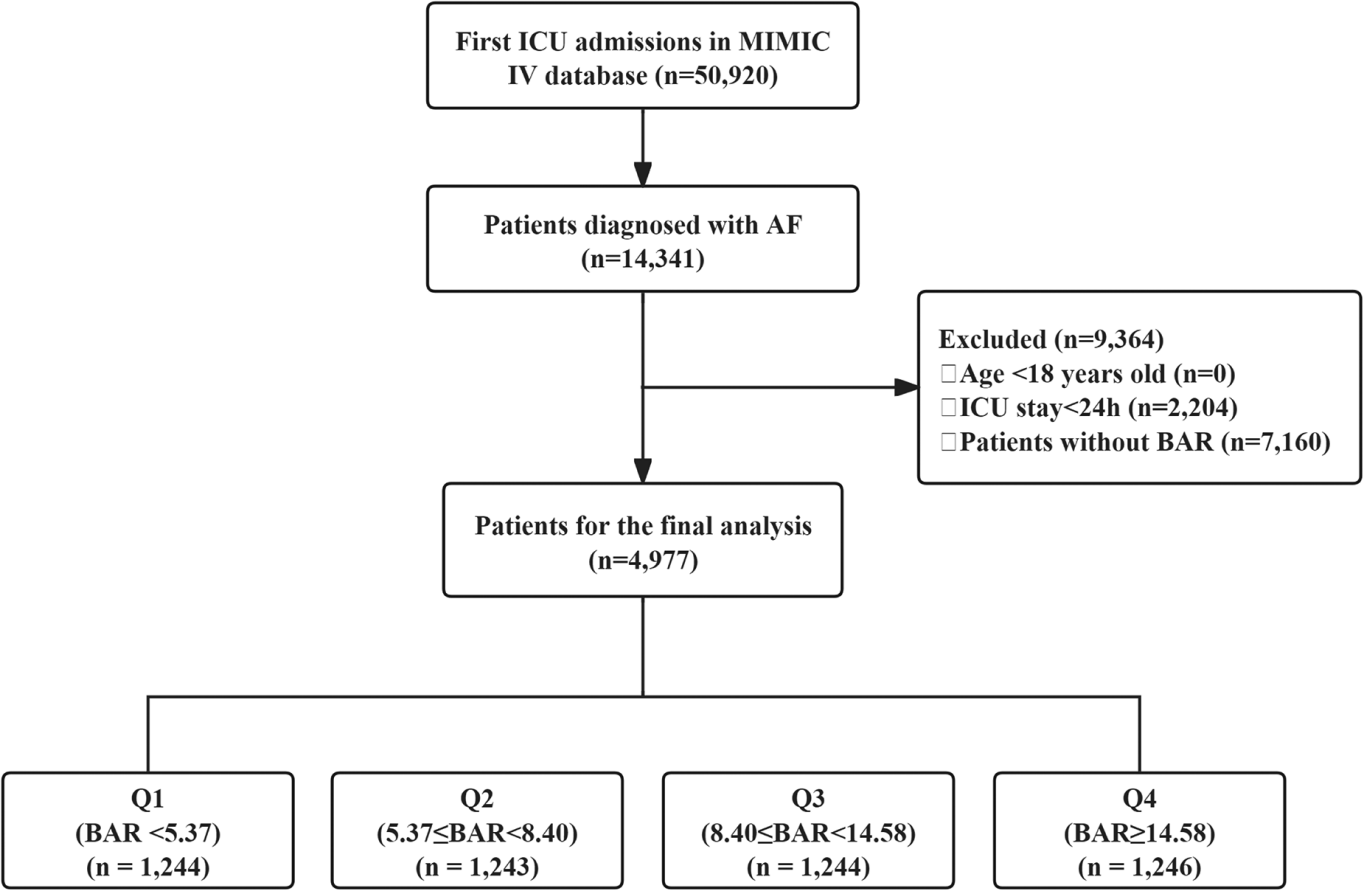
Flow chart of the study population. ICU, intensive care unit; MIMIC, medical information mart for intensive care IV; AF, atrial fibrillation; BAR, blood urea nitrogen to serum albumin ratio.

Adult patients (≥18 years) with a diagnosis of AF at their first ICU admission were included. Exclusion criteria were ICU stay <24 h and missing BUN or albumin data. Of the 14,341 eligible AF patients, 4,977 met the inclusion criteria (Figure 1). Patients were divided into BAR quartiles: Q1: BAR < 5.37 (*n* = 1,244); Q2: 5.37 ≤ BAR < 8.40 (*n* = 1,243); Q3: 8.40 ≤ BAR < 14.58 (*n* = 1,244); Q4: BAR ≥ 14.58 (*n* = 1,246).

### 2.3 Data extraction and BAR calculation

Patient data were extracted from the MIMIC-IV database using Structured Query Language (SQL). Collected variables included demographics (age, gender, race) and vital signs at ICU admission, such as heart rate, mean blood pressure (MBP), respiratory rate, and oxygen saturation (SpO₂). Illness severity was assessed using the Sequential Organ Failure Assessment (SOFA) score, Glasgow Coma Scale (GCS), and Charlson Comorbidity Index (CCI). Comorbidities such as coronary artery disease (CAD), heart failure, hypertension, obesity, diabetes, acute kidney injury (AKI), and sepsis were documented. Laboratory data, including white blood cell count (WBC), red cell distribution width (RDW), red blood cell count (RBC), platelet count, BUN, creatinine, potassium, sodium, albumin, international normalized ratio (INR), and N-terminal pro-brain natriuretic peptide (NT-proBNP), were collected. Therapeutic interventions, including the use of aspirin, heparin, warfarin, non-vitamin K antagonist oral anticoagulants (NOACs), amiodarone, statins, vasoactive drugs, ablation, mechanical ventilation, and continuous renal replacement therapy (CRRT), were also recorded. The BAR was calculated by dividing BUN (mg/dl) by serum albumin concentration (g/dl).

### 2.4 Outcomes

The primary outcomes were 28-day all-cause mortality. Secondary outcomes included length of hospital and ICU stay, hospital and ICU mortality.

### 2.5 Statistical analysis

Baseline characteristics were analyzed across BAR quartiles using appropriate statistical methods. Continuous variables were reported as mean ± standard deviation (SD) or median with interquartile range (IQR), depending on data distribution, while categorical variables were presented as frequencies and percentages. Comparisons between groups were performed using one-way analysis of variance (ANOVA) or the Kruskal–Wallis test for continuous variables, and the chi-square test for categorical variables, as applicable.

To identify factors associated with 28-day outcomes, we employed least absolute shrinkage and selection operator (LASSO) regression. Cox proportional hazards models were subsequently used to examine the relationship between BAR and 28-day all-cause mortality. To address potential confounding, we developed three models to calculate HRs and 95% CI, and performed trend analyses across quartiles. Model 1 adjusted for age, gender, and race. Model 2 incorporated Model 1 variables plus heart rate, respiratory rate, SpO_2_, SOFA score, GCS, CCI, CAD, AKI, and sepsis. Model 3 further adjusted for WBC, RDW, creatinine, albumin, INR, NT-proBNP, aspirin, warfarin, NOACs, statins, vasoactive drugs, and CRRT. RCS analysis was conducted to explore non-linear relationships.

Kaplan–Meier survival curves were used to compare primary outcomes across BAR quartiles. Additionally, receiver operating characteristic (ROC) curves were generated. Stratified and interaction analyses were performed based on factors such as age, gender, race, SOFA score, CAD, hypertension, AKI, sepsis, and NT-proBNP levels.

All analyses were conducted using R software (version 4.4.1) and Free Statistics software (version 2.0). Statistical significance was set at *P* < 0.05.

## 3 Results

### 3.1 Patient characteristics

Baseline characteristics of the study population by BAR quartiles are summarized in Table 1. The mean age was 74.4 ± 12.2 years, and males comprised 57.46% of the cohort. Both age and gender distribution varied significantly across quartiles (*P* < 0.001). Patients in higher BAR quartiles exhibited lower MBP and SpO_2_ (both *P* < 0.001), higher heart and respiratory rates (both *P* < 0.001), and elevated SOFA and CCI scores. They were also more likely to receive treatments such as heparin, vasoactive drugs, ventilatory support, and CRRT (all *P* < 0.001). Comorbidities, including heart failure, diabetes, AKI, and sepsis, were more prevalent in higher quartiles (all *P* < 0.001). Significant differences in laboratory parameters were observed across quartiles, including WBC, RDW, RBC, BUN, creatinine, potassium, sodium, albumin, INR, and NT-proBNP levels (all *P* < 0.001). Patients in higher quartiles also had longer hospital and ICU stays (both *P* < 0.001). In Quartile 4, hospital mortality reached 30.98%, ICU mortality was 20.87%, and 28-day mortality was 35.71% (all *P* < 0.001). Hospital and ICU stays were longest in patients with invasive ventilation, with median durations of 12.0 days (IQR: 7.2–20.7) and 5.8 days (IQR: 3.1–10.7), respectively (Supplementary Table S1).

**Table 1:** Baseline characteristics of the study participants.

Variables	Total	Q1	Q2	Q3	Q4	*P*
	(*n* = 4,977)	(BAR < 5.37)	(5.37 ≤ BAR < 8.40)	(8.40 ≤ BAR < 14.58)	(BAR ≥ 14.58)	
		(*n* = 1,244)	(*n* = 1,243)	(*n* = 1,244)	(*n* = 1,246)	
Age (year)	74.4 ± 12.2	71.7 ± 12.7	74.9 ± 12.0	75.7 ± 11.6	75.2 ± 11.9	<0.001
Gender, *n* (%)						<0.001
Female	2,117 (42.54)	602 (48.39)	548 (44.09)	525 (42.20)	442 (35.47)	
Male	2,860 (57.46)	642 (51.61)	695 (55.91)	719 (57.80)	804 (64.53)	
Race, *n* (%)						0.051
Other	1,439 (28.91)	386 (31.03)	324 (26.07)	363 (29.18)	366 (29.37)	
White	3,538 (71.09)	858 (68.97)	919 (73.93)	881 (70.82)	880 (70.63)	
Vital signs						
Heart rate (beats/min)	88 (75, 105)	86 (74, 103)	87 (74, 103)	90 (78, 108)	89 (75, 106)	<0.001
MBP (mmHg)	81 (69, 93)	85 (74, 97)	83 (71, 95.8)	78 (67, 90)	76 (66, 88)	<0.001
Respiratory rate (times/min)	19 (16, 23)	18 (15, 22)	19 (16, 23)	20 (16, 24)	20 (17, 24)	<0.001
SpO_2_ (%)	96.4 ± 4.4	97.1 ± 3.6	96.6 ± 4.2	95.9 ± 4.9	95.9 ± 4.7	<0.001
Severity scores						
SOFA	5 (3, 8)	3 (2, 5)	5 (3, 7)	5 (3, 8)	7 (5, 10)	<0.001
GCS	15 (14, 15)	15 (13, 15)	15 (14, 15)	15 (14, 15)	15 (13, 15)	0.227
CCI	6 (5, 8)	5 (4, 7)	6 (4, 8)	7 (5, 8)	8 (6, 9)	<0.001
Comorbidity						
CAD, *n* (%)	1,138 (22.87)	249 (20.02)	288 (23.17)	285 (22.91)	316 (25.36)	0.017
Heart failure, *n* (%)	2,518 (50.59)	425 (34.16)	607 (48.83)	702 (56.43)	784 (62.92)	<0.001
Hypertension, *n* (%)	2,051 (41.21)	462 (37.14)	525 (42.24)	513 (41.24)	551 (44.22)	0.003
Diabetes, *n* (%)	1,694 (34.04)	292 (23.47)	370 (29.77)	466 (37.46)	566 (45.43)	<0.001
AKI, *n* (%)	4,197 (84.33)	968 (77.81)	1,064 (85.60)	1,078 (86.66)	1,087 (87.24)	<0.001
Sepsis, *n* (%)	3,247 (65.24)	649 (52.17)	761 (61.22)	866 (69.61)	971 (77.93)	<0.001
Laboratory tests						
WBC (K/*μ*l)	11.0 (7.7, 15.7)	10.2 (7.5, 14.0)	10.9 (7.8, 15.3)	11.2 (7.8, 16.3)	11.7 (8.0, 17.6)	<0.001
RDW (%)	14.9 (13.8, 16.7)	14.1 (13.3, 15.3)	14.6 (13.7, 16.1)	15.2 (14.2, 16.9)	16.0 (14.7, 17.9)	<0.001
RBC (K/μl)	3.5 (3.0, 4.1)	3.8 (3.2, 4.3)	3.6 (3.1, 4.2)	3.5 (3.0, 3.9)	3.2 (2.8, 3.8)	<0.001
Platelet (K/μl)	187 (133, 253)	192 (139, 252)	190 (138, 250)	182 (132, 254)	183 (126, 257)	0.065
BUN (mg/dl)	26 (17, 42)	13 (11, 16)	21 (19, 24)	32 (28, 38)	61 (48, 79)	<0.001
Creatinine (mg/dl)	1.2 (0.8, 1.8)	0.8 (0.6, 1.0)	1.0 (0.8, 1.3)	1.4 (1.1, 1.8)	2.4 (1.7, 3.7)	<0.001
Potassium (mmol/L)	4.2 (3.8, 4.7)	4.0 (3.6, 4.4)	4.1 (3.8, 4.5)	4.3 (3.9, 4.7)	4.5 (4.0, 5.1)	<0.001
Sodium (mmol/L)	139 (135, 141)	139 (136, 141)	139 (136, 141)	138 (135, 141)	138 (134, 142)	<0.001
Albumin (g/dl)	3.1 (2.7, 3.5)	3.4 (3.0, 3.8)	3.2 (2.8, 3.6)	3.0 (2.6, 3.4)	2.8 (2.4, 3.2)	<0.001
INR	1.4 (1.2, 1.8)	1.3 (1.1, 1.6)	1.4 (1.2, 1.7)	1.4 (1.2, 1.9)	1.5 (1.3, 2.2)	<0.001
NT-proBNP (pg/ml), *n* (%)						<0.001
<2,000	832 (16.72)	232 (18.65)	231 (18.58)	203 (16.32)	166 (13.32)	
>=2,000	1,411 (28.35)	217 (17.44)	299 (24.05)	419 (33.68)	476 (38.20)	
Missing	2,734 (54.93)	795 (63.91)	713 (57.36)	622 (50.00)	604 (48.48)	
Treatment						
Aspirin, *n* (%)	2,070 (41.59)	542 (43.57)	581 (46.74)	483 (38.83)	464 (37.24)	<0.001
Heparin, *n* (%)	3,634 (73.02)	856 (68.81)	888 (71.44)	914 (73.47)	976 (78.33)	<0.001
Warfarin, *n* (%)	950 (19.09)	215 (17.28)	261 (21.00)	265 (21.30)	209 (16.77)	0.003
NOACs, *n* (%)	301 (6.05)	116 (9.32)	72 (5.79)	69 (5.55)	44 (3.53)	<0.001
Amiodarone, *n* (%)	1,337 (26.86)	298 (23.95)	359 (28.88)	348 (27.97)	332 (26.65)	0.033
Statin, *n* (%)	1,924 (38.66)	516 (41.48)	533 (42.88)	474 (38.10)	401 (32.18)	<0.001
Vasoactive drug, *n* (%)	2,467 (49.57)	488 (39.23)	624 (50.2)	649 (52.17)	706 (56.66)	<0.001
Ablation, *n* (%)	31 (0.62)	14 (1.13)	5 (0.40)	8 (0.64)	4 (0.32)	0.048
Ventilator						<0.001
No	720 (14.47)	226 (18.17)	162 (13.03)	171 (13.75)	161 (12.92)	
Non-invasive	2,044 (41.07)	508 (40.84)	485 (39.02)	516 (41.48)	535 (42.94)	
Invasive	2,213 (44.46)	510 (41.00)	596 (47.95)	557 (44.77)	550 (44.14)	
CRRT, *n* (%)	426 (8.56)	34 (2.73)	70 (5.63)	104 (8.36)	218 (17.5)	<0.001
Clinical outcomes						
Hospital stay (day)	10.0 (6.1, 16.8)	8.8 (5.8, 14.8)	9.8 (6.1, 16.5)	10.1 (6.2, 17.6)	11.6 (6.7, 18.9)	<0.001
ICU stay (day)	3.4 (2.0, 6.7)	2.9 (1.8, 5.5)	3.4 (1.9, 6.3)	3.5 (2.0, 6.9)	3.9 (2.2, 7.7)	<0.001
Hospital mortality, *n* (%)	980 (19.69)	129 (10.37)	178 (14.32)	287 (23.07)	386 (30.98)	<0.001
ICU mortality, *n* (%)	648 (13.02)	89 (7.15)	113 (9.09)	186 (14.95)	260 (20.87)	<0.001
28-day mortality, *n* (%)	1,144 (22.99)	158 (12.7)	210 (16.89)	331 (26.61)	445 (35.71)	<0.001

MBP, mean blood pressure; SpO_2_, oxygen saturation; SOFA, sequential organ failure assessment; GCS, Glasgow coma scale; CCI, Charlson comorbidity index; CAD, coronary artery disease; AKI, acute kidney injury; WBC, white blood cell; RDW, red cell distribution width; RBC, red blood cell; BUN, blood urea nitrogen; INR, international normalized ratio; NT-proBNP, N-terminal pro-brain natriuretic peptide; NOACs, Non-vitamin K antagonist oral anticoagulants; CRRT, continuous renal replacement therapy; ICU, intensive care unit.

### 3.2 Baseline characteristics of the 28-day survivor and mortality groups

The baseline characteristics of the study population, stratified by 28-day survival and mortality groups, are shown in Table 2. Patients in the mortality group were older 76.7 ± 12.0 (*P* < 0.001), and exhibited higher heart rates, respiratory rates, SOFA scores, and BAR levels 11.61 (*P* < 0.001). AKI and sepsis were more prevalent in the mortality group (*P* < 0.001). Laboratory findings revealed higher BUN, creatinine, potassium, and RDW, alongside lower serum albumin. Treatment differences included greater use of vasoactive drugs and CRRT in the mortality group, while aspirin and statin use was lower (*P* < 0.001).

**Table 2:** Baseline characteristics of the 28-day survivor and 28-day mortality group.

Variables	Total	28-day survival	28-day mortality	*P*
	(*n* = 4,977)	(*n* = 3,833)	(*n* = 1,144)	
Age (year)	74.4 ± 12.2	73.7 ± 12.1	76.7 ± 12.0	<0.001
Gender, *n* (%)				0.01
Female	2,117 (42.54)	1,594 (41.59)	523 (45.72)	
Male	2,860 (57.46)	2,239 (58.41)	621 (54.28)	
Race, *n* (%)				<0.001
Other	1,439 (28.91)	1,047 (27.32)	392 (34.27)	
White	3,538 (71.09)	2,786 (72.68)	752 (65.73)	
Vital signs				
Heart rate (beats/min)	88 (75, 105)	87 (74, 104)	92 (77, 108)	<0.001
MBP (mmHg)	81 (69, 93)	81 (70, 93)	79 (67, 93)	0.02
Respiratory rate (times/min)	19 (16, 23)	19 (16, 23)	20 (17, 24)	<0.001
SpO_2_ (%)	96.4 ± 4.4	96.6 ± 4.0	95.8 ± 5.4	<0.001
Severity scores				
SOFA	5 (3, 8)	5 (3, 7)	7 (4, 10)	<0.001
GCS	15 (14, 15)	15 (14, 15)	15 (12, 15)	<0.001
CCI	6 (5, 8)	6 (4, 8)	7 (6, 9)	<0.001
Comorbidity				
CAD, *n* (%)	1,138 (22.87)	850 (22.18)	288 (25.17)	0.034
Heart failure, *n* (%)	2,518 (50.59)	1,916 (49.99)	602 (52.62)	0.12
Hypertension, *n* (%)	2,051 (41.21)	1,612 (42.06)	439 (38.37)	0.03
Diabetes, *n* (%)	1,694 (34.04)	1,293 (33.73)	401 (35.05)	0.41
AKI, *n* (%)	4,197 (84.33)	3,148 (82.13)	1,049 (91.70)	<0.001
Sepsis, *n* (%)	3,247 (65.24)	2,342 (61.10)	905 (79.11)	<0.001
Laboratory tests				
WBC (K/μl)	11.0 (7.7, 15.7)	10.7 (7.6, 15.0)	12.1 (8.3, 18.1)	<0.001
RDW (%)	14.9 (13.8, 16.7)	14.7 (13.7, 16.3)	15.7 (14.3, 17.7)	<0.001
RBC (K/μl)	3.5 (3.0, 4.1)	3.5 (3.0, 4.1)	3.4 (2.9, 4.0)	<0.001
Platelet (K/μl)	187 (133, 253)	187 (135, 250)	188 (125, 262)	0.29
BUN (mg/dl)	26 (17, 42)	24 (16, 39)	34 (22, 53)	<0.001
Creatinine (mg/dl)	1.2 (0.8, 1.8)	1.1 (0.8, 1.7)	1.4 (0.9, 2.3)	<0.001
Potassium (mmol/L)	4.2 (3.8, 4.7)	4.2 (3.8, 4.6)	4.3 (3.8, 4.8)	<0.001
Sodium (mmol/L)	139 (135, 141)	139 (136, 141)	138 (135, 142)	0.69
Albumin (g/dl)	3.1 (2.7, 3.5)	3.2 (2.8, 3.6)	3.0 (2.5, 3.4)	<0.001
BAR	8.40 (5.37, 14.58)	7.60 (5.00, 12.94)	11.61 (7.20, 19.68)	<0.001
INR	1.4 (1.2, 1.8)	1.4 (1.2, 1.8)	1.5 (1.2, 2.1)	<0.001
NT-proBNP (pg/ml), *n* (%)				<0.001
<2,000	832 (16.72)	692 (18.05)	140 (12.24)	
>=2,000	1,411 (28.35)	1,096 (28.59)	315 (27.53)	
missing	2,734 (54.93)	2,045 (53.35)	689 (60.23)	
Treatment				
Aspirin, *n* (%)	2,070 (41.59)	1,659 (43.28)	411 (35.93)	<0.001
Heparin, *n* (%)	3,634 (73.02)	2,721 (70.99)	913 (79.81)	<0.001
Warfarin, *n* (%)	950 (19.09)	854 (22.28)	96 (8.39)	<0.001
NOACs	301 (6.05)	261 (6.81)	40 (3.50)	<0.001
Amiodarone, *n* (%)	1,337 (26.86)	1,012 (26.40)	325 (28.41)	0.18
Statin, *n* (%)	1,924 (38.66)	1,569 (40.93)	355 (31.03)	<0.001
Vasoactive drug, *n* (%)	2,467 (49.57)	1,733 (45.21)	734 (64.16)	<0.001
Ablation, *n* (%)	31 (0.62)	28 (0.73)	3 (0.26)	0.08
Ventilator				<0.001
No	720 (14.47)	579 (15.11)	141 (12.33)	
Non-invasive	2,044 (41.07)	1,675 (43.7)	369 (32.26)	
Invasive	2,213 (44.46)	1,579 (41.19)	634 (55.42)	
CRRT, *n* (%)	426 (8.56)	238 (6.21)	188 (16.43)	<0.001

MBP, mean blood pressure; SpO_2_, oxygen saturation; SOFA, sequential organ failure assessment; GCS, glasgow coma scale; CCI, Charlson comorbidity index; CAD, coronary artery disease; AKI, acute kidney injury; WBC, white blood cell; RDW, red cell distribution width; RBC, red blood cell; BUN, blood urea nitrogen; BAR, blood urea nitrogen to albumin ratio; INR, international normalized ratio; NT-proBNP, N-terminal pro-brain natriuretic peptide; NOACs, Non-vitamin K antagonist oral anticoagulants; CRRT, continuous renal replacement therapy.

### 3.3 Association between BAR and 28-day all-cause mortality

LASSO regression identified 26 variables for adjustment (Supplementary Figure S1). Multivariable Cox models (Table 3) confirmed BAR as an independent predictor of 28-day mortality. Each unit increase in BAR was associated with a 2% higher mortality risk (HR: 1.02; 95% CI: 1.01–1.03). Compared with Q1, patients in Q4 had an HR of 1.78 (95% CI: 1.42–2.22; *P* for trend < 0.001).

**Table 3:** Cox proportional hazard model assessing 28-day mortality in patients with AF.

Variables	Model 1		Model 2		Model 3	
	HR (95% CI)	*P*	HR (95% CI)	*P*	HR (95% CI)	*P*
28-day mortality						
BAR (continuous)	1.03 (1.03, 1.04)	<0.001	1.01 (1.01, 1.02)	<0.001	1.02 (1.01, 1.03)	<0.001
BAR (quartiles)						
Q1 (BAR < 5.37)	1 (ref)		1 (ref)		1 (ref)	
Q2 (5.37 ≤ BAR < 8.40)	1.33 (1.08, 1.64)	0.007	1.00 (0.81, 1.23)	0.996	1.01 (0.82, 1.25)	0.914
Q3 (8.40 ≤ BAR < 14.58)	2.19 (1.81, 2.65)	<0.001	1.40 (1.16, 1.73	0.001	1.51 (1.23, 1.85)	<0.001
Q4 (BAR ≥ 14.58)	3.18 (2.65, 3.82)	<0.001	1.63 (1.34, 2.00)	<0.001	1.78 (1.42, 2.22)	<0.001
*P* for trend		<0.001		<0.001		<0.001

Model 1: adjusted for age, gender and race.

Model 2: adjusted for Model 1 plus heart rate, MBP, respiratory rate, SpO_2_, SOFA, GCS, CCI, CAD, AKI, sepsis.

Model 3: adjusted for Model 2 plus WBC, RDW, creatinine, albumin, INR, NT-proBNP, aspirin, warfarin, NOACs, statin, vasoactive drug, CRRT.

MBP, mean blood pressure; SpO_2_, oxygen saturation; SOFA, sequential organ failure assessment; GCS, Glasgow coma scale; CCI, Charlson comorbidity index; CAD, coronary artery disease; AKI, acute kidney injury; WBC, white blood cell; RDW, red cell distribution width; INR, international normalized ratio; NT-proBNP, N-terminal pro-brain natriuretic peptide; NOACs, Non-vitamin K antagonist oral anticoagulants; CRRT, continuous renal replacement therapy.

### 3.4 Kaplan–Meier survival curve and RCS

**Figure 2:**
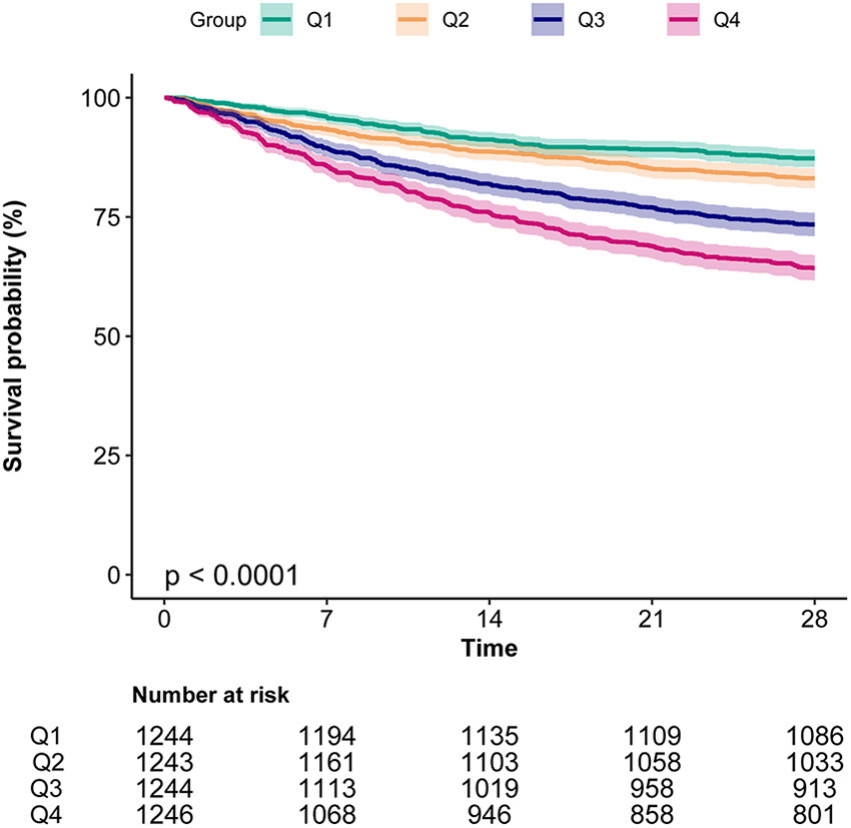
Kaplan–Meier curves indicates the association between the BAR and 28-day mortality of patients with AF. BAR, blood urea nitrogen to albumin ratio; AF, atrial fibrillation.

**Figure 3:**
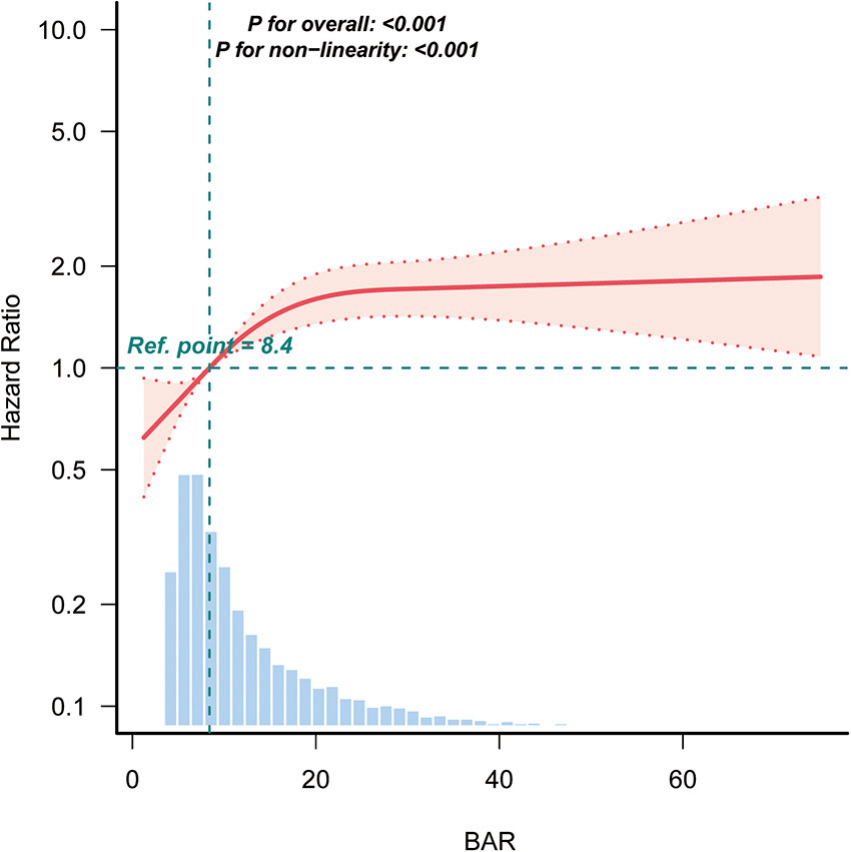
Restricted cubic spline analysis of the association between BAR and the risk of 28-day mortality in patients with AF. BAR, blood urea nitrogen to albumin ratio; AF, atrial fibrillation.

Kaplan–Meier curves (Figure 2) demonstrated significantly lower survival in patients with higher BAR levels (log-rank *P* < 0.0001), with Q4 showing the steepest decline. RCS analysis revealed a significant non-linear association between BAR and 28-day mortality (Figure 3).

### 3.5 Prediction of 28-day all-cause mortality by BAR

**Figure 4:**
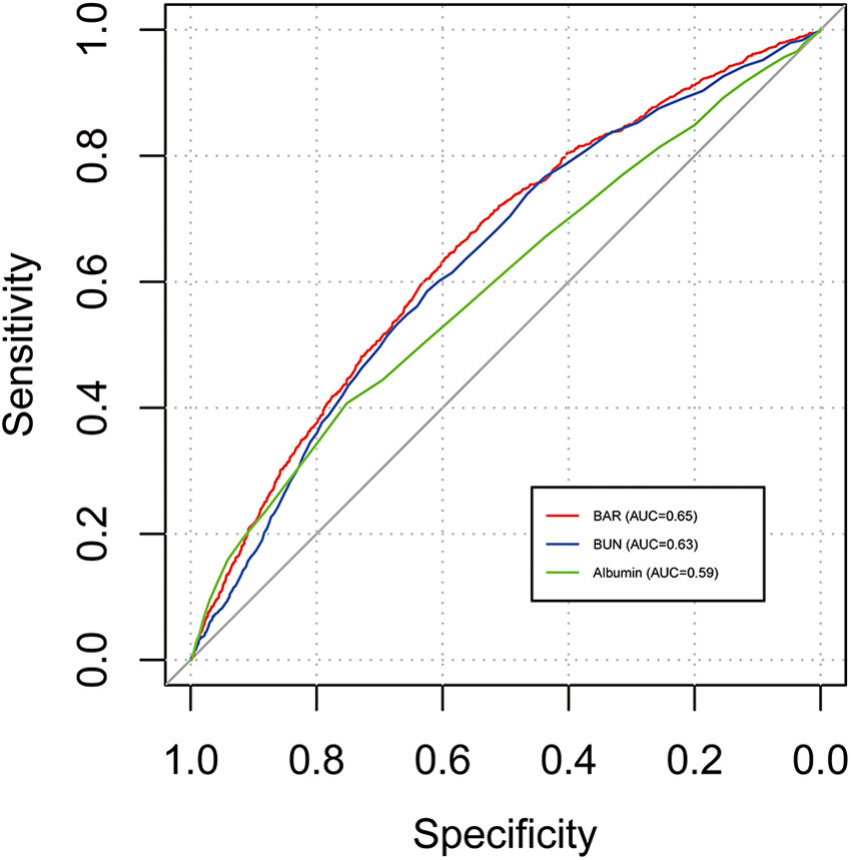
ROC curves of BAR for 28-day mortality of patients with AF. ROC, receiver operating characteristic; BAR, blood urea nitrogen to albumin ratio; AF, atrial fibrillation.

The ROC curves compare the predictive performance of BAR, BUN, and albumin (Table 4 and Figure 4). BAR had the highest AUC for 28-day mortality (AUC = 0.65), indicating superior predictive ability.

**Table 4:** Performance of BAR in predicting 28-day mortality in AF patients.

Variables	Cut-off	Sensitivity	Specificity	PPV	NPV	AUC (95% CI)
BAR	9.04	0.64	0.59	0.85	0.32	0.65 (0.63–0.67)
BUN	29.5	0.58	0.62	0.83	0.32	0.63 (0.62–0.65)
Albumin	2.75	0.41	0.75	0.67	0.19	0.59 (0.57–0.61)

BAR, blood urea nitrogen to albumin ratio; BUN, blood urea nitrogen; PPV, positive predictive value; NPV, negative predictive value; AUC, area under the curve.

### 3.6 Subgroup analysis

**Figure 5:**
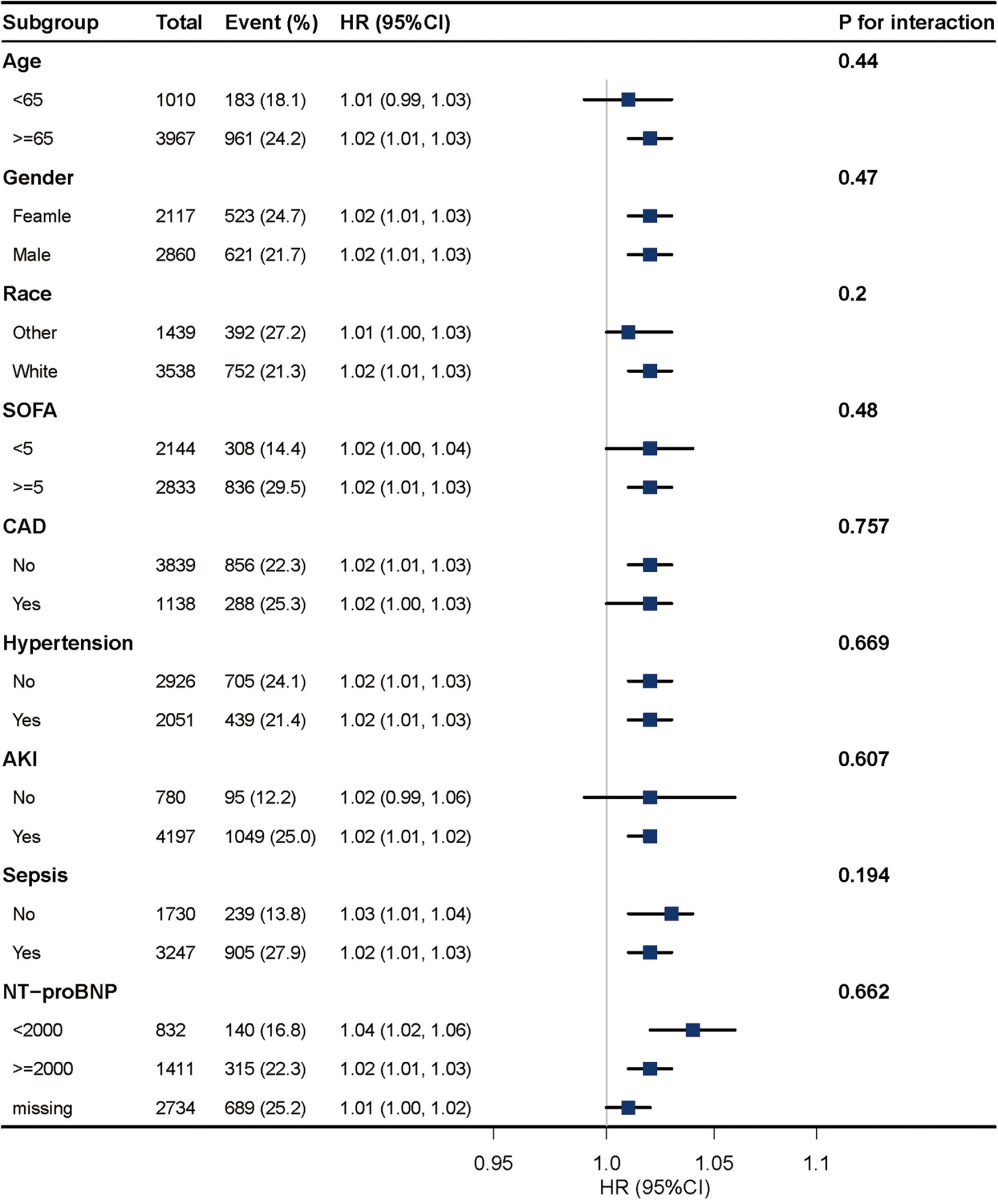
Association between BAR and 28-day mortality according to baseline characteristics. Each stratification was adjusted for all factors of Model 3 in Table 3 except for the stratification factor itself.

Subgroup analysis indicates no significant interactions for age, gender, race, SOFA score, CAD, hypertension, AKI, sepsis, and NT-proBNP (all *P* for interaction > 0.05) (Figure 5).

## 4 Discussion

This study demonstrates that the BAR is independently associated with 28-day mortality in critically ill patients with AF. The association remained significant after adjusting for demographic, clinical, and laboratory factors. Compared to BUN or albumin alone, BAR showed superior prognostic performance, supporting its utility as a simple and accessible biomarker for early risk stratification.

In critically ill patients with AF, BUN and albumin levels are important prognostic indicators. Elevated BUN typically indicates renal impairment or fluid retention (20, 21), both of which are closely associated with the onset and persistence of AF. Increased BUN levels lead to fluid accumulation, raising cardiac workload, promoting atrial dilation, and contributing to atrial fibrosis—all of which create a substrate for AF (22, 23). Moreover, elevated BUN is often associated with electrolyte imbalances, such as hyperkalemia, which can disrupt the heart's electrical function, increase the risk of arrhythmias, and prolong AF duration, thus worsening cardiac function and prognosis (20, 24). On the other hand, low albumin levels generally indicate chronic illness, malnutrition, or impaired liver function (25). As a plasma protein, albumin plays a critical role in maintaining colloid osmotic pressure and transporting drugs and nutrients. Low albumin levels suggest nutritional depletion or severe illness, leading to increased vascular permeability, edema, and fluid shifts. Additionally, hypoalbuminemia may indicate an underlying inflammatory state (26), which can accelerate cardiac and organ failure, reducing survival rates.

BAR serves as an effective marker of this complex interplay, providing a comprehensive measure of disease severity. Elevated BAR levels in AF patients often signal multiorgan dysfunction, including renal and hepatic impairment, which is common in critically ill patients (27, 28). Moreover, BAR has been associated with increased thromboembolic risk and atrial remodeling. High BUN levels reflect systemic stress and a prothrombotic state (13), while low albumin correlates with reduced anticoagulant efficacy and increased embolic risk (29). Unlike isolated biomarkers, BAR offers a holistic assessment of the metabolic, hemodynamic, and inflammatory shifts that characterize AF, making it a practical tool for risk stratification and treatment planning, especially in high-risk patients.

Although BAR was independently associated with 28-day mortality, its discriminatory power was moderate, with an AUC of 0.65. However, BAR showed better predictive performance compared with its individual components, with an AUC of 0.63 for BUN and 0.59 for albumin. This suggests that BAR may provide a more integrated assessment of renal function and nutritional status. These findings support its potential role as a composite prognostic biomarker. Nevertheless, due to its limited sensitivity and specificity, BAR may not be sufficient as a standalone risk stratification tool. Further research is needed to assess whether incorporating BAR into established risk prediction models, such as CHA_2_DS_2_-VASc or HAS-BLED, could improve prognostic accuracy and inform clinical decision-making in patients with atrial fibrillation.

Notably, our findings revealed relatively low rates of anticoagulant use, particularly in patients with high BAR levels (Q4 group), where warfarin was used in only 16.77% of cases and NOACs in just 3.53%. Current guidelines recommend oral anticoagulation as standard therapy for patients with atrial fibrillation, especially those with persistent or permanent AF at elevated thromboembolic risk. However, our dataset lacked detailed information on AF duration or classification, precluding analysis of whether the patients had newly diagnosed or chronic AF. The underuse of guideline-directed medical therapy (GDMT), including anticoagulants, may partially explain the higher mortality observed in the high-BAR group. Inadequate anticoagulation could contribute to increased thromboembolic complications or progression of atrial remodeling, compounding the risk associated with renal dysfunction and hypoalbuminemia. Future studies should incorporate detailed rhythm history and therapeutic adherence data to better delineate these associations.

In our study based on the MIMIC-IV database, the median ICU length of stay among intubated patients was 5.8 days (IQR: 3.1–10.7), which appears relatively short. Several factors may account for this. First, early ICU mortality was common in our cohort and could contribute to shorter ICU stays. Second, MIMIC includes a substantial proportion of patients with treatment-limiting orders such as Do-Not-Resuscitate (DNR) or Do-Not-Intubate (DNI), which may lead to earlier withdrawal of intensive care. Third, hospital-specific discharge practices and ICU resource limitations may have led to early transitions to step-down or general wards. Similar ICU durations have also been reported in previous studies using the MIMIC-III and IV datasets (30, 31). Importantly, the relatively short ICU stay may have implications for our results. It could lead to underestimation of disease progression or complications that typically manifest later during prolonged ICU admissions. Moreover, the early timing of data collection may favor early predictors of mortality, while longer-term prognostic factors may be less represented. Therefore, while our findings remain valid within the context of early ICU outcomes, caution is warranted when generalizing them to patients with prolonged ICU courses.

This study has several strengths, including the use of a large, well-curated critical care database and rigorous statistical adjustment. However, limitations should be acknowledged. First, the retrospective design inherently introduces potential selection bias and limits the ability to draw causal inferences. Second, this study was based on data from the MIMIC-IV database, which represents a single-center ICU cohort in the United States. As such, the generalizability of our findings to other healthcare settings, geographic regions, or patient populations may be limited. Third, BAR may be affected by unmeasured confounders such as hydration status, liver function, and chronic malnutrition, which were not fully accounted for due to data limitations. Fourth, echocardiographic data were not available in the MIMIC-IV database, which limited our ability to assess cardiac structure and function, including left ventricular ejection fraction and atrial size. Lastly, our study did not compare BAR with established AF-specific risk scores (e.g., CHA_2_DS_2_-VASc, ATRIA, or HAS-BLED), which limits our ability to evaluate the incremental prognostic value of BAR beyond current tools. In addition, while MIMIC-IV is a valuable resource, analyses based on such databases require careful attention to case definitions, data quality, and residual confounding. For example, evaluating the effect of anticoagulation is challenging, as treated patients may differ systematically in comorbidity burden or disease severity. Without careful statistical adjustment, treatment effects may be confounded. Moreover, data on anticoagulation intensity and adherence are limited, and MIMIC-IV does not capture post-discharge outcomes. This constrains assessment of long-term endpoints such as stroke, bleeding, or readmission. External validation in prospective cohorts is needed to confirm these findings.

In conclusion, BAR is an independent and easily accessible predictor of short-term mortality in critically ill patients with AF. Its integration into routine clinical assessment may enhance early risk stratification and inform personalized treatment strategies in this vulnerable population.

## Data Availability

Publicly available datasets were analyzed in this study. These data are available from the MIMIC-IV 2.2 database at https://physionet.org/content/mimiciv/2.2/.
