# Supplementary material for: Blood urea nitrogen to serum albumin ratio as a prognostic marker for 28-day mortality in atrial fibrillation: a retrospective cohort study
Source: Front Cardiovasc Med. 2025 May 9;12:1533575. doi: 10.3389/fcvm.2025.1533575 (PMC12098446; doi:10.3389/fcvm.2025.1533575)
Supplement: Supplementary file 1 [file Datasheet1.pdf]

Table S1 Clinical outcomes stratified by ventilation status

Variables	Total (n = 4977)	No (n = 720)	Non-invasive (n = 2044)	Invasive (n = 2213)	*P*
Hospital stay (day)	10.0 (6.1, 16.8)	7.0 (4.3, 12.7)	9.1 (6.0, 14.7)	12.0 (7.2, 20.7)	< 0.001
ICU stay (day)	3.4 (2.0, 6.7)	2.0 (1.5, 3.3)	2.7 (1.8, 4.2)	5.8 (3.1, 10.7)	< 0.001
Hospital mortality, n (%)					< 0.001
No	3997 (80.31)	603 (83.75)	1798 (87.96)	1596 (72.12)	
Yes	980 (19.69)	117 (16.25)	246 (12.04)	617 (27.88)	
ICU mortality, n (%)					< 0.001
No	4329 (86.98)	648 (90.00)	1951 (95.45)	1730 (78.17)	
Yes	648 (13.02)	72 (10.00)	93 (4.55)	483 (21.83)	
28-day mortality, n (%)					< 0.001
No	3833 (77.01)	579 (80.42)	1675 (81.95)	1579 (71.35)	
Yes	1144 (22.99)	141 (19.58)	369 (18.05)	634 (28.65)	

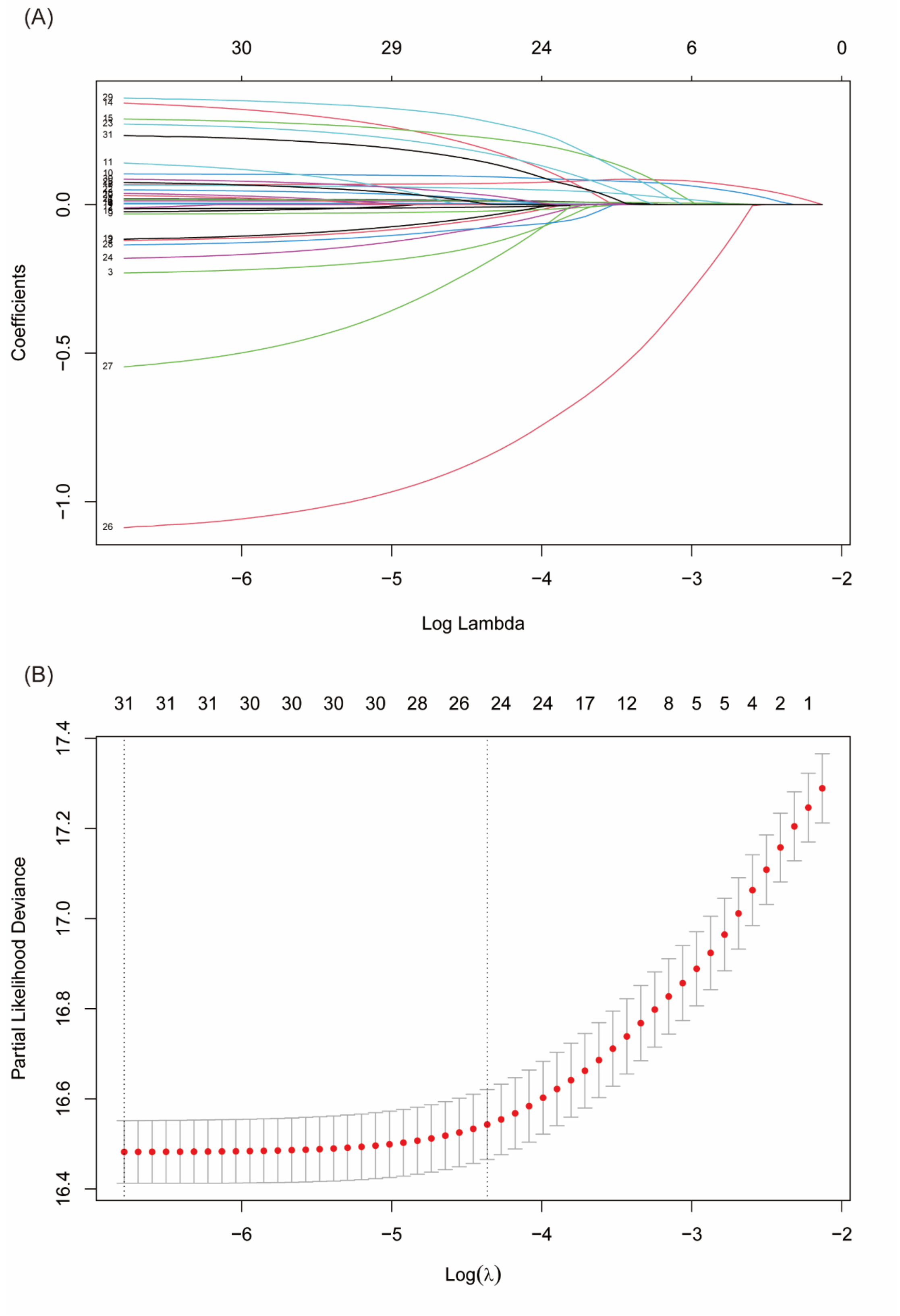

Figure S1 Selection process of prognostic variables of atrial fibrillation by least absolute shrinkage and selection operator regression. (B) Selection process of the value of lambda by cross-validation.
